# Single-Cell Gene Module Inference Reveals Alternative Polyadenylation Dynamics Associated with Autism

**DOI:** 10.3390/ijms27062849

**Published:** 2026-03-21

**Authors:** Fei Liu, Haoran Yang, Xiaohui Wu

**Affiliations:** 1 Cancer Institute, Suzhou Medical College, Soochow University, Suzhou 215000, China; l13139058980@163.com (F.L.); yanghr0307@163.com (H.Y.); 2 Suzhou Key Laboratory of Pathogen Bioscience and Anti-Infective Medicine, MOE Key Laboratory of Geriatric Diseases and Immunology, Suzhou Medical College, Soochow University, Suzhou 215000, China; 3 Jiangsu Key Laboratory of Infection and Immunity, Soochow University, Suzhou 215000, China; 4 Pediatric Hematology & Oncology Key Laboratory of Higher Education Institutions in Jiangsu Province, Suzhou 215000, China

**Keywords:** autism spectrum disorder, alternative polyadenylation, gene module, single-nucleus RNA sequencing

## Abstract

Autism spectrum disorder (ASD) is a neurodevelopmental condition characterized by genetic heterogeneity. Post-transcriptional regulation—particularly alternative polyadenylation (APA)—plays a critical role in the pathogenesis of ASD. APA controls mRNA stability, translational efficiency, and subcellular localization through modulating the length of the 3′ untranslated region of mRNA. APA profiling can uncover functionally relevant post-transcriptional alterations often missed by conventional gene expression analyses. However, current ASD analyses still largely rely on differential gene expression or individual APA event detection, which ignores the collective explanatory power of ASD risk genes or co-dysregulated functional gene modules within specific cell types. In this study, we present an integrative computational framework that combines matrix factorization and machine learning to identify ASD-associated gene modules driven by APA and to predict cell-type-specific ASD-related cells. Applied to human brain single-nucleus RNA sequencing (snRNA-seq) data, our approach systematically uncovers APA regulatory patterns that are specific to cell type, brain region, and sex in ASD. The identified APA modules are significantly enriched in pathways related to synaptic function, neurodevelopment, and immune response, with the strongest signals observed in excitatory neurons of the prefrontal cortex. Using APA genes from these modules as features, we built a classification model that effectively distinguishes ASD cells from normal cells. Moreover, we found that integrating APA with gene expression—two complementary modalities—substantially improves prediction accuracy, underscoring APA as an independent and biologically informative regulatory layer. Our work delineates a high-resolution APA regulatory landscape in ASD, offering novel insights and potential therapeutic avenues beyond transcriptional abundance.

## 1. Introduction

Autism spectrum disorder (ASD) is a complex neurodevelopmental disorder with a highly heterogeneous etiology, involving multiple genetic variations and closely related to immune dysregulation and dysfunction of the gut microbiota–brain axis [1,2,3,4,5]. With the advancement of genetic exploration, an increasing number of ASD susceptibility genes have been revealed, with a total of about a thousand records so far. Among them, the open directory maintained by the Simons Foundation Autism Research Initiative (SFARI) is considered a benchmark [6,7]. However, there is currently no single gene that can dominate the diagnosis, treatment, or phenotype prediction of ASD. Currently, gene expression analysis in ASD research has advanced to the single-cell level. Velmeshev et al. conducted the first large-scale single-nucleus RNA sequencing (snRNA-seq) study on cortical tissues from individuals with ASD and controls, revealing that transcriptional abnormalities in ASD are predominantly localized to upper-layer excitatory neurons and microglia [8]. Differentially expressed genes in these cell types are significantly enriched in functional pathways closely associated with core ASD phenotypes. Subsequently, Werling et al. further confirmed transcriptional alterations in upper-layer excitatory neurons and identified upregulation of genes related to microglial activation and neuroinflammation, implicating innate immune responses in the molecular pathogenesis of ASD [9]. Collectively, these snRNA-seq studies have systematically mapped cell-type-specific gene expression disruptions in ASD brain tissue, providing a high-resolution molecular landscape for understanding disease mechanisms.

In addition to gene expression changes, post-transcriptional regulation—particularly alternative polyadenylation (APA)—plays a critical role in the pathogenesis of ASD [10,11]. APA modulates the length of the 3′ untranslated region (3′ UTR) of mRNA, thereby influencing regulatory elements such as microRNA (miRNA) binding sites, RNA-binding protein (RBP) interactions, and N^6^-methyladenosine (m^6^A) modification sites, which collectively control mRNA stability, translational efficiency, and subcellular localization [12,13]. Given that these functions are largely determined by the 3′ UTR, APA profiling can uncover functionally relevant post-transcriptional alterations often missed by conventional gene expression analyses. For example, integrative analysis of brain tissues identified 286 APA-associated ASD genes, approximately 60–65% of which were undetected by expression-based methods and enriched in pathways related to intracellular transport and localization, suggesting that APA may impact neuronal function through regulation of mRNA spatial distribution [14]. Notably, gain-of-function mutations in the 3′ UTR are significantly enriched in individuals with ASD, potentially leading to 3′ UTR shortening, escape from miRNA-mediated repression, and consequent overexpression of neuronal genes [15]. Moreover, 3′ UTR abnormalities have been observed in neurodevelopmental disorders comorbid with ASD, such as fragile X syndrome (*FMR1*) and Rett syndrome (*MECP2*) [16], while neural RBPs like *Elavl3* can modulate poly(A) site selection to induce 3′ UTR lengthening and influence neurodevelopmental processes [17].

Although single-cell sequencing technologies have been widely applied in ASD research and have begun to reveal the potential role of APA in specific neuronal subtypes, current analyses still largely rely on gene-level differential expression or individual APA event detection [18,19]. Such approaches have notable limitations: on the one hand, the collective explanatory power of known ASD risk genes remains low [20]; on the other hand, at single-cell resolution, ASD-related pathological phenotypes are rarely driven by isolated genes but rather emerge from co-dysregulated functional gene modules within specific cell types [21,22]. This is particularly relevant for post-transcriptional regulatory mechanisms like APA, whose biological impact often manifests as coordinated changes in 3′ UTR length across a set of co-regulated genes, rather than significant alterations in any single gene, rendering such signals prone to being overlooked by conventional single-gene analytical frameworks.

In this study, we present an integrative computational framework that combines matrix factorization and machine learning to identify ASD-associated gene modules driven by APA and to predict cell-type-specific ASD-related cells. Applied to human brain snRNA-seq data, our approach systematically uncovers APA regulatory patterns that are specific to cell type, brain region, and sex in ASD. The identified APA modules are significantly enriched in pathways related to synaptic function, neurodevelopment, and immune response, with the strongest signals observed in excitatory neurons of the prefrontal cortex. Using APA genes from these modules as features, we built a classification model that effectively distinguishes ASD cells from normal cells. Moreover, we found that integrating APA with gene expression—two complementary modalities—substantially improves prediction accuracy, underscoring APA as an independent and biologically informative regulatory layer. Our work delineates a high-resolution APA regulatory landscape in ASD and reveals functionally coherent modules shaped by APA dysregulation. Our results highlight the critical role of post-transcriptional control in ASD pathogenesis, offering novel insights and potential therapeutic avenues beyond transcriptional abundance.

## 2. Results

### 2.1. Overview of the Pipeline

**Figure 1:**
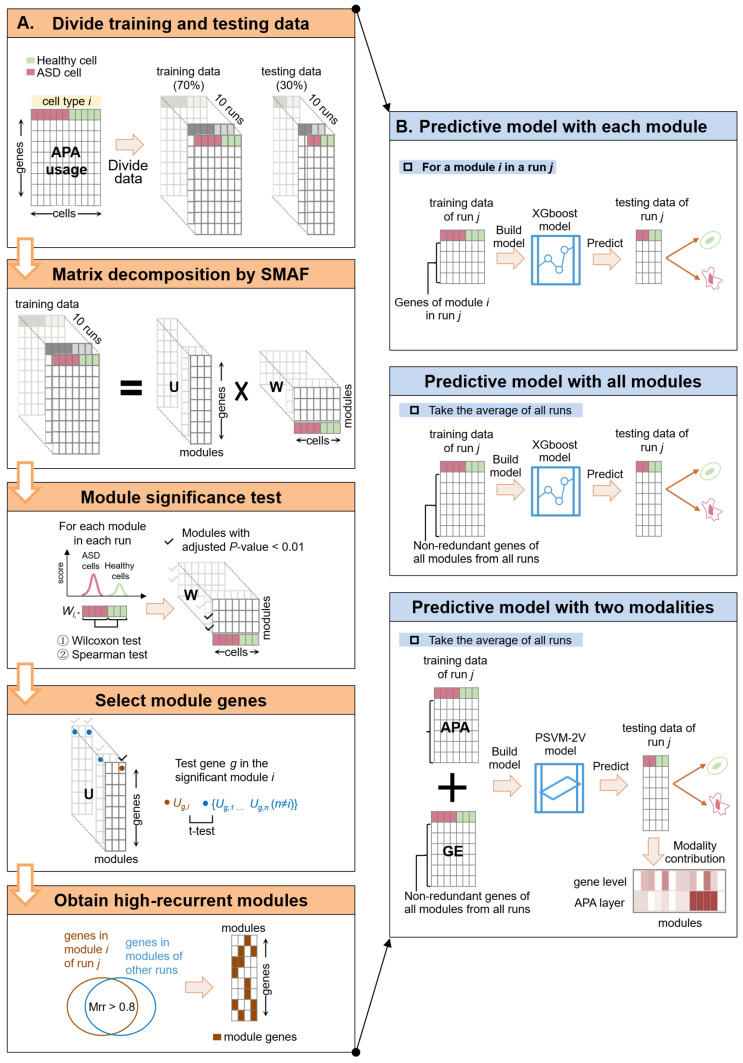
Schematic workflow of the pipeline. (**A**) The APA module identification mainly consists of five core steps: data partitioning, matrix decomposition, module evaluation, feature selection, and module selection. First, based on the APA usage matrix of each cell type, the data was randomly divided into a training set and a test set repeatedly. Next, sparse module activity factorization (SMAF) was used to perform matrix decomposition on the training set, obtaining a module–gene matrix (U) and a module–cell activity matrix (W). Subsequently, combined with statistical tests, modules that showed significant differences between the ASD and control groups and were highly correlated with phenotypes were retained. Then core genes and specific APA features in each module were identified. Finally, the stability of modules was evaluated based on the recurrent rate across data partitions, and highly stable modules that repeatedly appeared in multiple training sets were selected. (**B**) Construction of cell-type-specific ASD prediction models based on identified APA modules. Distinct cell-type-specific predictive models were built and evaluated using APA genes from individual APA modules or those from combined modules. Moreover, an integrated predictive model was also constructed using both modalities of APA modules and gene expression modules to strengthen the predictive power.

In this study, we constructed a systematic analysis pipeline aimed at identifying APA gene modules associated with ASD (Figure 1A) and constructing cell-type-specific ASD prediction models based on identified APA modules (Figure 1B). The APA module identification mainly consists of five core steps: data partitioning, matrix decomposition, module evaluation, feature selection, and module selection (Figure 1A). First, based on the APA usage matrix of each cell type, the data was randomly divided into a training set and a test set at a ratio of 7:3, and this process was repeated 10 times to enhance robustness. Next, sparse module activity factorization (SMAF) [23] was used to perform matrix decomposition on the training set, obtaining a module–gene matrix (U) and a module–cell activity matrix (W) to identify potential APA gene modules. Subsequently, combined with statistical tests, modules that showed significant differences between the ASD and control groups and were highly correlated with phenotypes were retained. Then core genes and specific APA features in each module were identified. Then, the stability of modules was evaluated based on the recurrent rate across data partitions, and highly stable modules that repeatedly appeared in multiple training sets were selected. After obtaining APA modules, cell-type-specific predictive models were constructed for ASD prediction (Figure 1B). Distinct predictive models were built and evaluated using APA genes from individual APA modules or those from combined modules. Moreover, an integrated predictive model was also constructed using both modalities of APA modules and gene expression modules to strengthen the predictive power.

### 2.2. Genome-Wide APA Profile Distinguishes Cell Types in ASD

**Figure 2:**
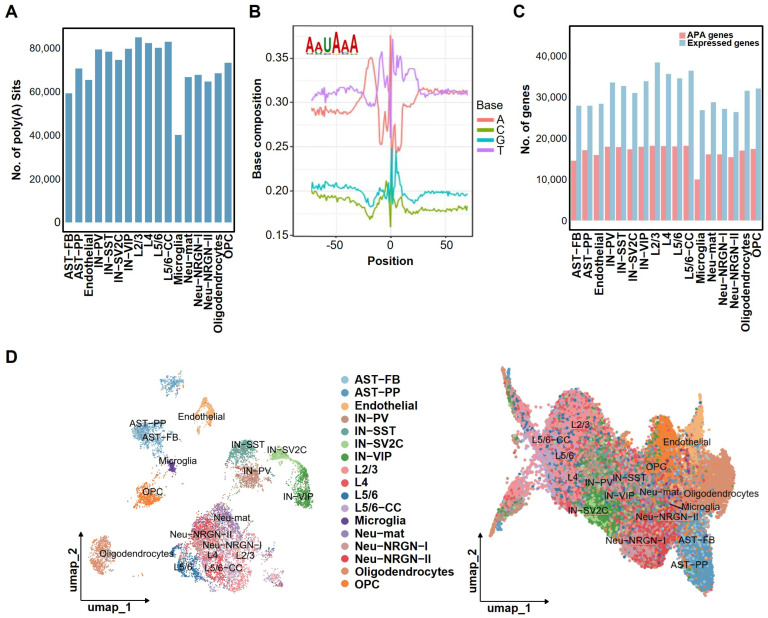
Identification of 3′ UTR poly(A) sites and cell type clustering based on APA profiles. (**A**) Distribution of identified 3′ UTR poly(A) sites across different cell types, revealing that microglia exhibit the lowest number of detected sites compared to other lineages. (**B**) Quality control validation showing the nucleotide frequency surrounding poly(A) cleavage sites and the significant enrichment of the canonical polyadenylation signal (AATAAA) within the upstream region, confirming the high reliability of site identification. (**C**) Comparison of the total number of expressed genes versus APA-regulated genes across cell types, highlighting the heterogeneity in APA gene coverage among different brain cell populations. (**D**) UMAP dimensionality reduction visualizations demonstrating that both the poly(A) site expression profile (**left**) and the APA usage profile (**right**) measured by the RUD (relative usage of the distal poly(A) site) effectively segregate single nuclei into distinct major brain cell type clusters, indicating strong cell-type-specific APA regulatory patterns.

We collected human brain snRNA-seq data from the previous study [8] (Table S1) and identified genome-wide poly(A) sites at the single-nucleus level. A total of 99,307 poly(A) sites located within 3′ UTR regions were obtained (Table S2). The number of 3′ UTR poly(A) sites for each cell type in ASD was further summarized (Figure 2A), revealing microglia as the cell type with the fewest identified poly(A) sites. To validate the accuracy of the identified poly(A) sites, we examined the nucleotide distribution surrounding the sites and the presence of canonical polyadenylation signals (PAS) (Figure 2B). The most typical PAS motif, AATAAA, was significantly enriched within 50 nucleotides upstream of the poly(A) sites, confirming the reliability of the site identification. The number of expressed APA genes and total genes varied across different cell types (Figure 2C).

UMAP (uniform manifold approximation and projection) was applied to the poly(A) site profiles, the APA usage matrix measured by the RUD score, and the gene expression matrix for dimensionality reduction, clustering, and visualization (Figure 2D and Figure S1). The results demonstrate that both poly(A) site expression and RUD profiles can clearly separate single cells into distinct major brain cell types. Major cell types formed relatively independent clusters in the UMAP plots, indicating cell-type-specific APA regulatory patterns. Although the gene expression-based UMAP also distinguished major cell types, its clustering performance and degree of cell type separation differed from those observed in the APA-based UMAP visualizations.

### 2.3. Identification of ASD-Related APA Gene Modules with Cell Type Specificity

**Figure 3:**
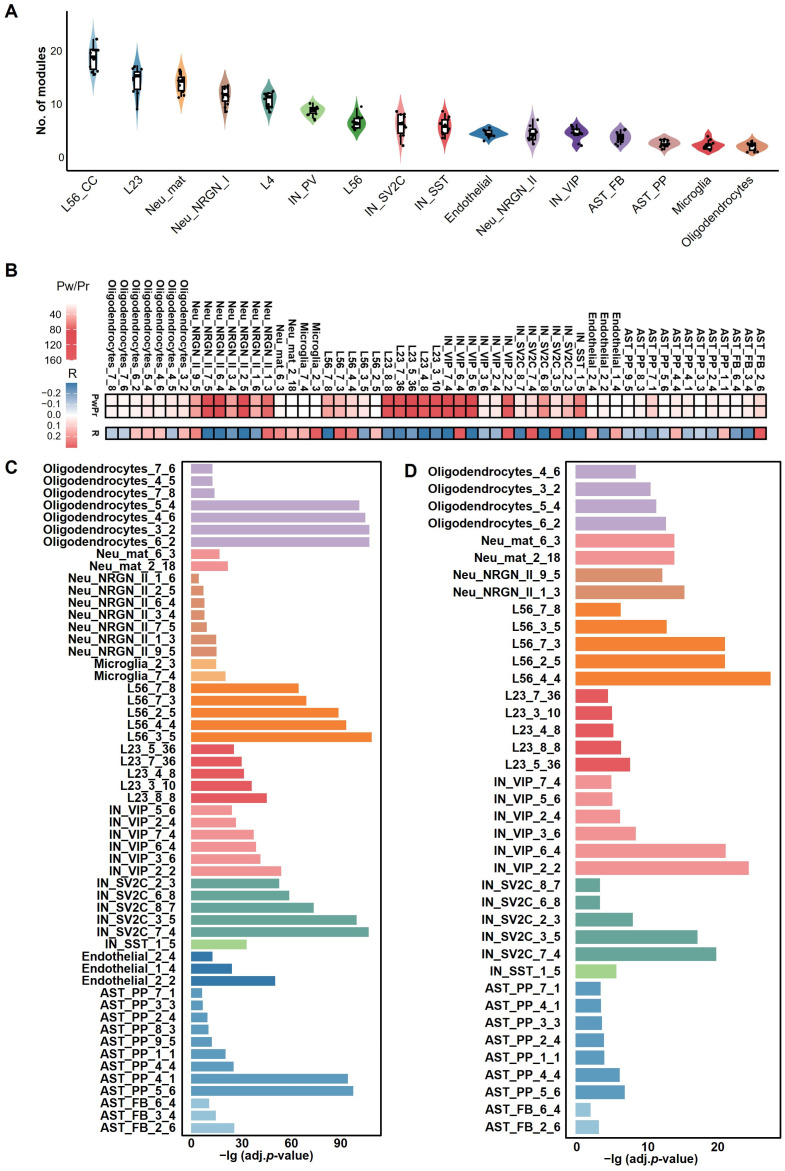
Analysis of ASD-associated APA gene modules across cell types. (**A**) Distribution of ASD-associated APA gene modules across cell types, revealing significant heterogeneity. Excitatory neurons, particularly the L5/6-CC subtype, consistently exhibit the highest number of ASD-related modules across all training sets, suggesting their pivotal role in APA dysregulation in ASD. The violin plot displays the module count distribution per cell type, with each dot representing a single training set. (**B**) Statistical significance and robustness of the 55 highly recurrent ASD-associated APA modules (recurrence rate > 80%). Rows “Pw” and “Pr” show the −lg adjusted *p*-value from Wilcoxon rank-sum and Spearman correlation tests, respectively; row “R” shows the Spearman correlation coefficient. Each APA gene module is labeled by a combination of cell type, data partition ID, and module ID within that partition (e.g., L2/3_1_1 denotes the first module identified in the first training set of L2/3 cells). (**C**) Functional enrichment analysis of genes in the 55 high-recurrent APA modules with cell-type-specific marker genes. (**D**) Functional enrichment analysis of genes in the 55 high-recurrent APA modules with known ASD risk genes from the SFARI database.

By applying SMAF to ten training sets for each cell type, we identified gene modules reflecting coordinated APA regulatory programs and poly(A) site usage preferences [23]. Through two complementary statistical screening strategies (see Methods), a set of APA modules significantly associated with ASD was identified, revealing substantial heterogeneity across cell types. The analysis revealed marked differences in the number of ASD-associated APA gene modules among cell types (Figure 3A), reflecting the cellular heterogeneity underlying ASD. Specifically, excitatory neurons—including subtypes L2/3, L4, L5/6, and L5/6-CC—contained a significantly higher number of ASD-related modules compared to other cell types. Notably, the L5/6-CC consistently exhibited the highest number of ASD-associated APA modules across all training sets. This distribution pattern suggests a potentially pivotal role of excitatory neurons, particularly the L5/6-CC subtype, in APA dysregulation in ASD. This finding aligns with the study by Velmeshev et al. [8], which similarly reported that synaptic signaling in upper-layer excitatory neurons is particularly vulnerable in ASD and that molecular alterations in L5/6-CC neurons are more closely associated with clinical manifestations of ASD.

To mitigate uncertainty arising from random initialization in the SMAF matrix factorization process, we introduced the recurrent rate as a metric for assessing module robustness, aiming to identify highly stable modules (see Methods). We identified 55 highly recurrent ASD-associated APA gene modules. These modules not only passed the aforementioned significance tests (Figure 3B) but also demonstrated high consistency across data partitions and decomposition runs, indicating high robustness and biological interpretability. These modules were primarily derived from excitatory neurons (L2/3 and L5/6), inhibitory neurons (IN-VIP and IN-SV2C), and neurons expressing neurogranin (*NRGN*), suggesting that these cell types exhibit more pronounced and consistently reproducible APA dysregulation in ASD, potentially playing critical roles in its pathogenic mechanisms.

To validate the cell-type specificity of the APA gene modules and their association with ASD, gene enrichment analyses were performed. Hypergeometric tests revealed that all 55 modules were significantly enriched for marker genes of their corresponding cell types (Figure 3C), with modules from L5/6 showing the highest enrichment, underscoring their strong cell type specificity. Further analysis showed that 39 of these modules were significantly enriched for ASD risk genes from the SFARI database, most of which originated from L5/6 neurons (Figure 3D), highlighting the central role of this neuronal subtype in ASD pathogenesis. In contrast, modules derived from endothelial cells and microglia did not show significant enrichment for SFARI genes and were thus excluded from downstream analyses.

Additionally, we analyzed functional relationships among modules by computing Spearman correlation coefficients between modules based on the U matrix. Results showed high correlations among modules from the same or similar cell types (Figure S2), particularly among those derived from L5/6 neurons, suggesting their involvement in shared biological processes or regulatory networks. This result further supports the cell type specificity of the modules and provides insights into their potential functional roles in ASD.

### 2.4. Cell-Type-Specific APA Dynamics in ASD

**Figure 4:**
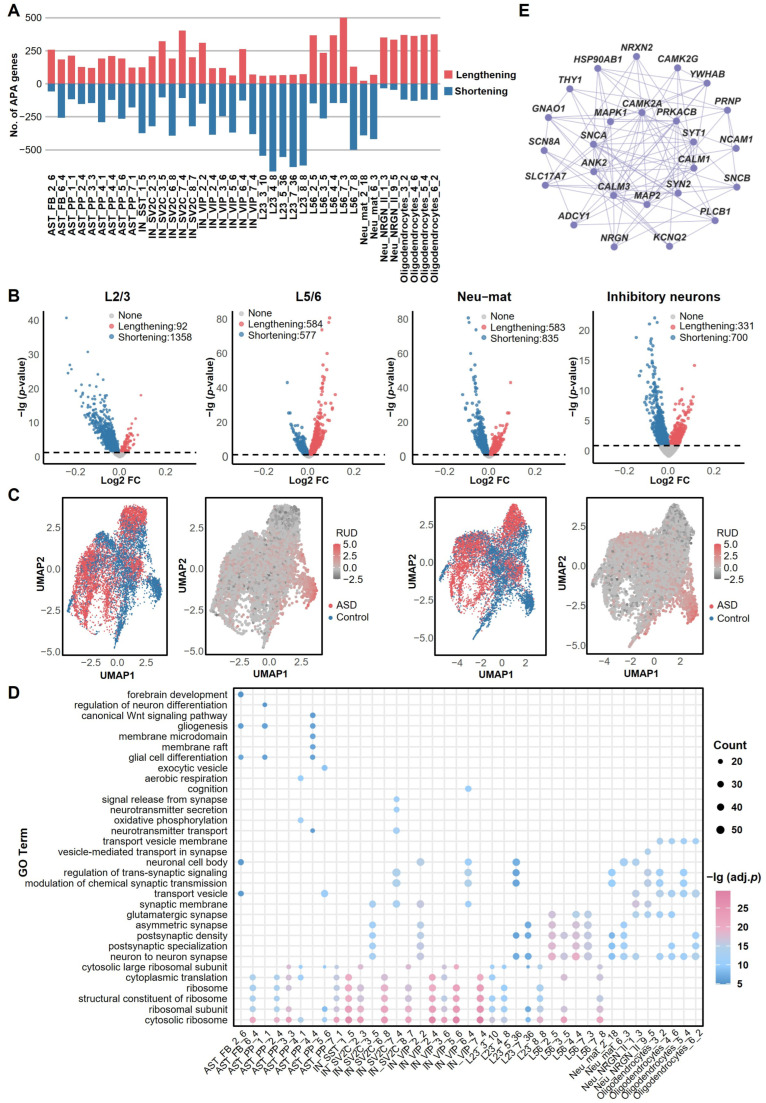
Cell-type-specific APA alterations in ASD. (**A**) The number of 3′ UTR lengthening and shortening events between ASD and control groups across 39 ASD-associated APA modules significantly enriched for SFARI genes. Significant APA abnormalities are most prominent in L2/3 and L5/6 excitatory neurons, Neu-NRGN-II neurons, and oligodendrocytes, highlighting these cell types as key sites of dysregulation. (**B**) Trends in 3′ UTR length changes in APA genes in L2/3, L5/6, mature neurons (Neu-mat), and all inhibitory neurons. Notably, contrasting trends in 3′ UTR length changes among neuronal subtypes. L2/3 neurons predominantly exhibit 3′ UTR shortening (proximal site usage), whereas L5/6 neurons show significant lengthening (distal site usage). (**C**) Changes in APA patterns in two representative modules within L2/3. UMAP visualization showing the 2D embeddings of ASD and control cells based on the APA profiles, as well as the RUD score of cells in module L2/3_4_8 (left two panels). The right two panels show results for the L2/3_5_36 module. The distinct clustering patterns observed in these modules highlight cell state-specific APA alterations in ASD. (**D**) GO functional enrichment analysis of genes in each APA module. (**E**) Protein–protein interaction (PPI) network for the L5/6_2_5 module, identifying critical hub genes (e.g., *MAPK1*, *CAMK2A*, *SYT1*, *CALM1*, *PRNP*) involved in synaptic function, calcium signaling, and neuronal development.

We conducted a systematic analysis of APA site selection for 39 cell-type-specific APA modules enriched for SFARI genes. Results revealed significant abnormalities in APA site selection in multiple neuronal subtypes and glial cells in individuals with ASD, most prominently in L2/3 excitatory neurons, L5/6 excitatory neurons, Neu-*NRGN*-II neurons, and oligodendrocytes (Figure 4A and Figure S3). Notably, although both L2/3 and L5/6 are excitatory neurons, they exhibit opposing APA regulatory trends (Figure 4B): L2/3 neurons predominantly favor proximal poly(A) site usage, leading to 3′ UTR shortening, whereas L5/6 neurons preferentially utilize distal poly(A) sites, resulting in 3′ UTR lengthening. This contrasting APA pattern suggests distinct post-transcriptional regulatory mechanisms may be involved in different excitatory neuronal subtypes. Additionally, widespread 3′ UTR shortening is observed in inhibitory neurons, indicating potential broad dysregulation within their post-transcriptional regulatory networks. A previous study based on conventional differential expression analyses did not find significant changes in gene expression profiles of Neu-mat [8]. However, our APA analysis uncovered a substantial number of differential APA events in this cell type (Figure 4B). Specifically, 1589 genes exhibited significant APA alterations in ASD, with 1377 of them showing a preference for proximal poly(A) site usage. Our results suggest that APA dysregulation could be a key contributor to ASD pathogenesis.

UMAP analysis further revealed significant cell-type-specific changes in APA patterns between ASD patients and controls (Figure 4C). In multiple modules, cells from the ASD and control groups showed clear separation based on poly(A) site usage, particularly pronounced in the L2/3_4_8 module, indicating a specific alteration in APA regulation within this module in ASD. Functional analysis demonstrated that APA modules are significantly enriched in key neurobiological processes such as regulation of neuron differentiation and synaptic signaling (Figure 4D). Excitatory neuron modules are primarily involved in synaptic plasticity and Wnt signaling pathway regulation, while inhibitory neuron modules are enriched for neurotransmitter secretion and regulation of membrane potential (Figure S4). KEGG (Kyoto Encyclopedia of Genes and Genomes) pathway analysis further revealed significant enrichment of APA gene modules in multiple neural-related pathways, including glutamatergic synapses, GABAergic synapses, calcium signaling, and long-term potentiation (Figure S5A). Moreover, pathway network analysis illustrated tight interconnections among these pathways, particularly forming a complex regulatory network centered on neurodegenerative diseases, multiple diseases, and glutamatergic synapses. Notably, the interaction between long-term potentiation and the calcium signaling pathway was especially prominent and most evident in the L5/6 layer (Figure S5B). These findings suggest that APA regulation may contribute to the onset and progression of ASD by affecting multiple critical signaling pathways and their coordinated interactions.

To gain deeper insights into the molecular characteristics of APA modules, we analyzed the top 10 genes with the most significant APA changes in ASD versus controls (Figure S6A) and presented their intersections with SFARI genes and cell type markers (Figure S6B). The results revealed that several genes, including *SLC1A3* and *GLUL* (glutamate metabolism), *SLC6A1* (GABA transport), and *SYT1* and *ATP1B1* (synaptic transmission), exhibit significant APA alterations in ASD, suggesting they may be key targets of APA dysregulation.

Based on the PPI (protein–protein interaction) network constructed from APA modules, several hub genes that repeatedly appeared in multiple networks were identified, such as *MAPK1*, *CAMK2A*, *SYT1*, *CALM1*, and *PRNP* (Figure 4E). These genes are primarily enriched in critical pathways related to synaptic function, calcium signaling, and neuronal development and are closely associated with ASD phenotypes [24,25,26,27,28]. For instance, *SYT1*, *NRXN2*, *NCAM1*, and *CALM1* have been documented in the literature to be tightly linked to synaptic plasticity and neurodevelopment [29,30,31,32]. Furthermore, modules from different cell types exhibit distinct functional preferences: excitatory neuron modules are enriched for synaptic plasticity-related genes, while inhibitory neuron modules are associated with neurotransmitter signaling (Figure 4E and Figure S7).

In summary, APA dysregulation associated with ASD is not only present in multiple neuronal subtypes but also broadly affects glial cells, which may contribute to disease pathogenesis by disrupting synaptic function, post-transcriptional regulation, and key signaling pathways.

### 2.5. Sex-Specific APA Regulation Reveals Differential Mechanisms in Synaptic and Metabolic Pathways Between Males and Females in ASD

**Figure 5:**
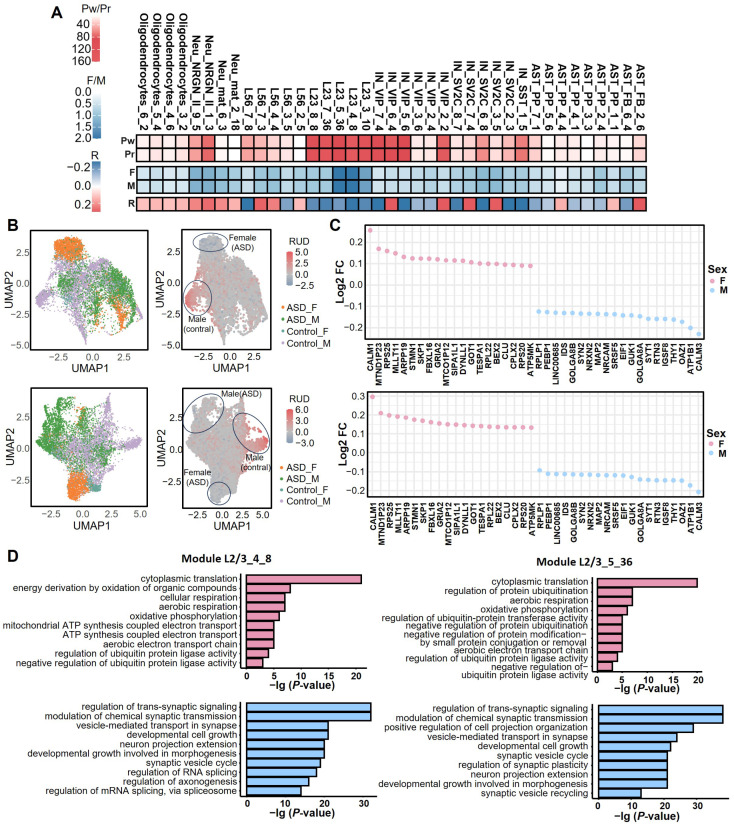
Sex-specific APA regulatory patterns in ASD. (**A**) Associations between APA gene modules and sex (F/M) phenotypes. Rows “Pw” and “Pr” show the −lg adjusted *p*-value from Wilcoxon rank-sum and Spearman correlation tests, respectively; row “R” shows the Spearman correlation coefficient; rows “F” and “M” display association measured by FCA scores between APA modules and sex phenotype (F, female; M, male). The strong color intensity for modules L2/3_4_8 and L2/3_5_36 indicates a significant association of the two modules with sex. Modules L2/3_3_10 and L2/3_5_36 exhibit the strongest associations with sex, consistently verified across all statistical metrics. (**B**) UMAP visualization showing the 2D embeddings of male and female ASD and control cells based on the APA profiles, as well as the RUD score of cells in module L2/3_4_8 (upper two panels). The lower two panels show results for the L2/3_5_36 module. The cell cluster outlined by the ellipse exhibits significant APA pattern differences under the interaction of sex and diagnostic status, suggesting sex-specific regulation of poly(A) site selection in ASD. (**C**) Top 20 genes associated with female and male cells identified through DEAPA analysis in modules L2/3_3_10 and L2/3_5_36. log_2_FC > 0 indicates a stronger association with females (enriched in metabolic genes), while log_2_FC < 0 indicates a stronger association with males (enriched in synaptic genes). (**D**) GO enrichment analysis revealing distinct functional mechanisms: female-associated genes are primarily enriched in energy metabolism pathways (e.g., oxidative phosphorylation), whereas male-associated genes are concentrated in synaptic functions (e.g., neurite extension and synaptic transmission), suggesting a metabolic compensation mechanism in females versus synaptic vulnerability in males.

ASD has been consistently shown to exhibit significant sex differences, with a markedly higher prevalence in males than in females [33,34]. In this study, the FCA (factor–covariate association) metric of the sciRED algorithm (see Methods) was used to analyze the correlation between 39 ASD-related cell-type-specific APA modules and sex (Figure 5A) [35]. The results showed that the L2/3_3_10 and L2/3_5_36 modules had high association scores with sex, and this finding was consistently verified by the Wilcoxon test and Spearman correlation analysis.

UMAP visualization demonstrated distinct differences in poly(A) site usage patterns between males and females, both in ASD cases and controls, for these two modules (Figure 5B). Further differential APA analysis identified genes with significant sex-specific changes (Figure 5C), followed by GO (Gene Ontology) functional enrichment analysis (Figure 5D). In the L2/3_3_10 module, genes associated with females were primarily enriched in energy metabolism processes, such as oxidative phosphorylation and mitochondrial ATP synthesis, whereas male-associated genes were concentrated in synaptic functions, including neurite extension and regulation of chemical synaptic transmission. This suggests that females may employ enhanced mitochondrial metabolism as a compensatory mechanism, while males are more susceptible to synaptic dysfunction, potentially explaining their higher ASD incidence.

Moreover, key synaptic genes such as *SYT1*, *SYN2*, and *SNAP25* in males exhibited a preference for proximal poly(A) site usage, resulting in 3′ UTR shortening. This alteration may attenuate miRNA-mediated repression of their mRNAs, thereby enhancing translational efficiency and potentially contributing to synaptic hyperexcitability—a core pathophysiological feature of ASD. In contrast, in females, mitochondrial genes such as *NDUFA4*, *COX6C*, and *ATP5F1* showed a preference for distal poly(A) sites, which might enhance miRNA repression and lead to reduced expression, thereby impacting energy metabolism [36]. Analysis of the L2/3_5_36 module also revealed sex-specific differences: the regulation of the ubiquitin-protein ligase activity pathway was significantly activated in females, potentially mitigating neuroinflammation through enhanced protein degradation [37,38], whereas in males, dysregulation of RNA splicing may disrupt the generation of splice isoforms critical for synaptic plasticity.

In summary, APA modulates synaptic and metabolic pathways through sex-specific poly(A) site selection. Males appear more vulnerable to synaptic dysfunction, while females may achieve phenotypic compensation by enhancing mitochondrial metabolism or proteostasis. These findings provide a potential mechanistic explanation for the observed sex bias in ASD.

### 2.6. Brain Region-Specific APA Regulation Drives Phenotypic Heterogeneity in ASD

**Figure 6:**
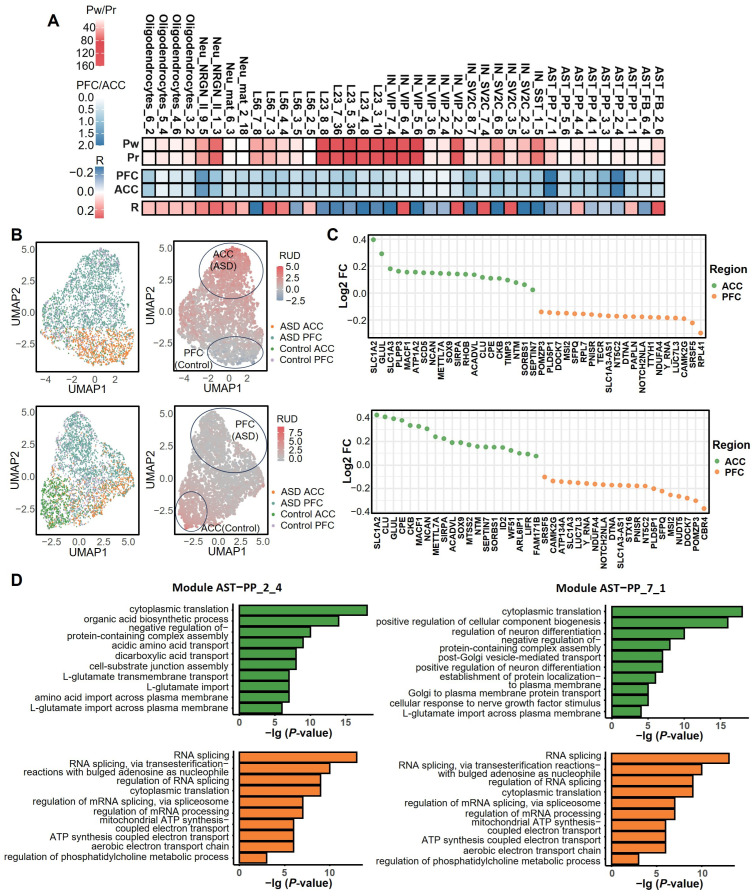
Functional divergence of APA modules in two brain regions, ACC and PFC. (**A**) Associations between APA gene modules and brain region (ACC/PFC) phenotypes. Rows “Pw” and “Pr” show the −lg adjusted *p*-value from Wilcoxon rank-sum and Spearman correlation tests, respectively; row “R” shows the Spearman correlation coefficient; rows “PFC” and “ACC” display the association measured by FCA scores between APA modules and region phenotype. The strong color intensity for modules AST-PP_2_4 and AST-PP_7_1 indicates a significant association of these two modules with the brain region. Modules AST-PP_2_4 and AST-PP_7_1 exhibit the strongest associations with brain region, indicating significant region-specific APA dysregulation in ASD. (**B**) UMAP visualization showing the 2D embeddings of PFC and ACC ASD and control cells based on the APA profiles, as well as the RUD score of cells in module AST-PP_2_4 (upper two panels). The lower two panels show results for the AST-PP_7_1 module. The cell cluster outlined by the ellipse exhibits significant APA pattern differences under the interaction of brain region and diagnostic status, suggesting brain region-specific regulation of poly(A) site selection in ASD. (**C**) Top 20 genes associated with ACC and PFC, respectively, identified through DEAPA analysis in modules AST-PP_2_4 and AST-PP_7_1. log_2_FC > 0 indicates stronger enrichment in ACC (associated with glutamate homeostasis and transport), while log_2_FC < 0 indicates stronger enrichment in PFC (associated with synaptic transmission). (**D**) GO enrichment analysis revealing distinct functional mechanisms: ACC-associated genes are primarily enriched in glutamate homeostasis, amino acid transport, and neuroinflammation-related pathways, whereas PFC-associated genes are concentrated in glutamatergic synaptic transmission and oxidoreductase activity. This suggests APA dysregulation in the ACC may contribute to social behavioral deficits, while alterations in the PFC aggravate cognitive impairment.

Multiple studies have consistently demonstrated structural and functional abnormalities in key brain regions in individuals with ASD, including the prefrontal cortex (PFC), temporal cortex, anterior cingulate cortex (ACC), and amygdala—regions critically involved in social cognition and neurodevelopment [39,40,41,42,43]. Here, we found that two modules, AST-PP_2_4 and AST-PP_7_1, exhibited a significant difference between the brain regions ACC and PFC (Figure 6A). These two modules were significantly associated with ASD in a region-specific manner, suggesting that APA exerts brain region-specific regulatory patterns in individuals with ASD (Figure 6B). Differential APA analysis identified genes most strongly associated with each brain region within the AST-PP_2_4 and AST-PP_7_1 modules (Figure 6C). Functional enrichment analysis revealed that differentially regulated APA genes in the AST-PP_2_4 module associated with the ACC were enriched in functions related to glutamate homeostasis, such as cytoplasmic translation and dicarboxylic acid transport (Figure 6D). For example, *SLC1A2* and *CLU* undergo 3′ UTR lengthening through distal poly(A) site selection, potentially enhancing mRNA stability to maintain glutamate homeostasis in the ACC, thereby influencing the regulation of emotional and social behaviors [44]. In contrast, PFC-associated genes were primarily enriched in glutamatergic synaptic transmission functions; *SLC1A3*, for instance, tends to use proximal poly(A) sites, leading to 3′ UTR shortening, which may reduce miRNA-mediated repression and enhance synaptic excitability—closely linked to cognitive impairments in the PFC [45].

The AST-PP_7_1 module further supports region-specific APA regulation. This module showed high activity in the ACC, where its APA-regulated genes were enriched in functions related to blood–brain barrier transport and transmembrane amino acid transport (Figure 6D). For example, *CPE* and *NCAN* utilize distal poly(A) sites to extend their 3′ UTRs, potentially modulating the secretion efficiency of neurotrophic factors and influencing neuroinflammatory processes [46]. In the PFC, genes in this module were primarily enriched in oxidoreductase activity; *DTNA* and *NOTCH2NLA* tend to use proximal poly(A) sites, which may disrupt the assembly of postsynaptic density protein networks, impair synaptic plasticity, and exacerbate deficits in cognitive flexibility [47].

In summary, APA drives phenotypic heterogeneity in ASD by region-specific mRNA processing in the ACC and PFC, regulating key molecular networks at the post-transcriptional level. APA dysregulation in the ACC primarily involves disturbances in amino acid metabolism and neuroinflammation, potentially contributing to social behavioral deficits. In contrast, APA alterations in the PFC focus on glutamatergic signaling pathways and oxidative stress damage, further aggravating cognitive impairment [44].

### 2.7. Integrated Analysis of Cell-Type-Specific ASD Prediction Models Based on APA and Gene Expression Profiles

**Figure 7:**
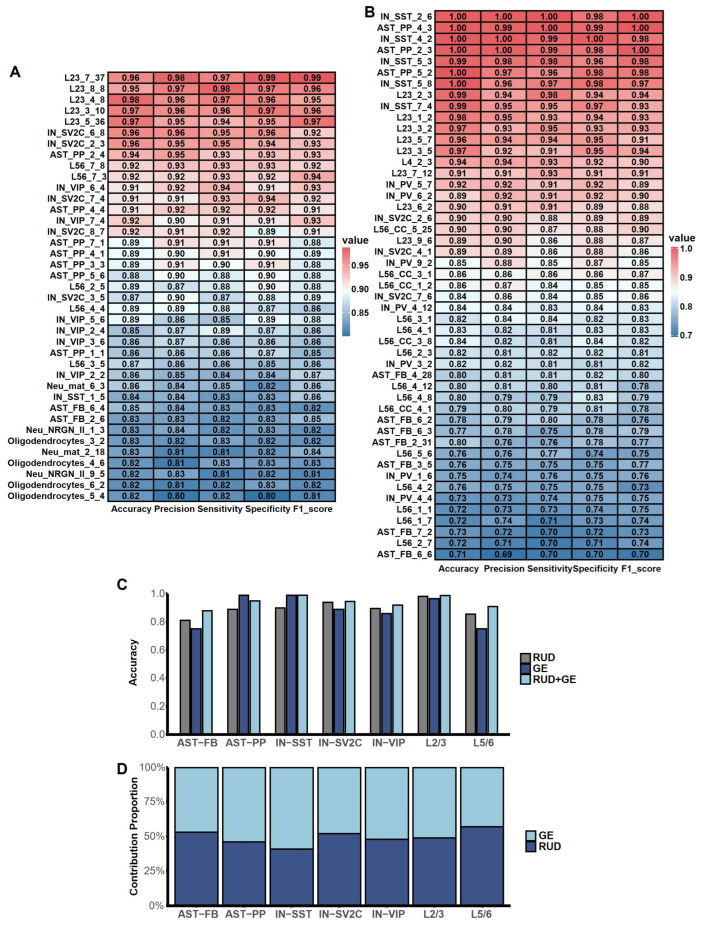
Cell-type-specific ASD prediction based on APA and gene expression profiles. (**A**) Performance of cell-type-specific ASD prediction models constructed using 39 APA modules across nine cell types, highlighting significant predictive power in neurons and glial cells. (**B**) Performance of cell-type-specific ASD prediction models constructed using 49 gene expression modules. Notably, GE modules identified additional types (L4, L5/6-CC, IN-VIP) but failed to detect modules in Neu-NRGN-II and oligodendrocytes, which were captured by APA analysis. (**C**) Comparison of prediction accuracy among models based on APA modules (RUD), gene expression (GE) modules, and integrated modules (RUD + GE) across seven shared cell types present in both the APA and gene expression profiles. The single-modality prediction model based on RUD or GE was constructed using the XGBoost classifier, while the model integrating the two modalities was built using the PSVM-2V classifier. (**D**) Contribution of the APA and gene expression modality for the prediction model.

To evaluate the predictive power of the APA gene modules for ASD, we constructed cell-type-specific prediction models using the eXtreme Gradient Boosting (XGBoost) classifier (Figure 7A). For comparison between APA and gene expression data, we applied the same analytical framework to the gene expression matrix to identify 49 cell-type-specific gene modules associated with ASD (Figure 7B and Figure S8). These gene modules spanned nine cell types: L2/3, L4, L5/6, L5/6-CC, IN-SST, IN-PV, IN-SV2C, AST-FB, and AST-PP, predominantly derived from neurons and glial cells, reaffirming the critical roles of these two cell classes in ASD pathogenesis. Compared to the APA modules, the gene expression modules included additional cell types—L4, L5/6-CC, and IN-VIP—but failed to identify modules in Neu-*NRGN*-II and oligodendrocytes. This discrepancy suggests that transcriptome-level analyses alone may overlook molecular abnormalities in certain cell types, whereas APA analysis can uncover latent APA dysregulation and its association with ASD in Neu-*NRGN*-II and oligodendrocytes.

We further examined the overlap between genes identified in APA modules and gene expression modules for shared cell types (Figure S9). The results revealed that, in cell types such as IN-SV2C, IN-VIP, and L5/6, the APA modules include a significantly larger number of genes than their gene expression-based counterparts, highlighting the unique advantage of APA analysis in revealing cell-type-specific associations with ASD.

Moreover, to assess the performance differences between APA and gene expression modules in ASD prediction, we built unimodal classification models using XGBoost for each modality and calculated prediction accuracy across seven shared cell types (AST-FB, AST-PP, IN-SST, IN-SV2C, IN-VIP, L2/3, and L5/6) (Figure 7C). The results showed that, in AST-FB, IN-SV2C, and L5/6, models based on APA modules outperformed those based on gene expression modules in the same cell types. This indicates that, for specific cell types, APA dysregulation may play a more critical role in ASD than transcriptional changes, suggesting that APA alterations could be more central to the pathological mechanisms underlying neurodevelopmental and functional impairments in these cell types.

To exploit the complementary information from APA and gene expression data and improve prediction performance, we performed a multimodal fusion analysis using the Multi-view Privileged Support Vector Machine (PSVM-2V) model. For each shared cell type, we took the union of genes from APA and gene expression modules, built XGBoost models on both the RUD matrix and the gene expression matrix, and compared prediction accuracy before and after fusion (Figure 7C). We also estimated the contribution of each modality for the prediction (Figure 7D). The results demonstrated that, in the PSVM-2V multimodal model, prediction accuracy improved to varying degrees in all cell types except AST-PP, with particularly notable gains in cell types that initially showed lower performance, such as AST-FB and L5/6. Furthermore, the RUD matrix generally contributed more to the multimodal model, indicating that APA data provides unique and valuable information for ASD prediction. Our results showed that APA regulation not only holds significant implications for understanding ASD pathogenesis but also offers a novel and informative dimension for constructing disease prediction models.

## 3. Discussion

ASD is a highly heterogeneous neurodevelopmental condition whose molecular mechanisms remain incompletely understood. Growing evidence indicates that APA, a pivotal post-transcriptional regulatory mechanism, may play a critical role in the pathogenesis of ASD. The unique regulatory resolution provided by APA data presents a powerful opportunity to advance conventional gene expression and gene module analyses. In this study, leveraging human brain snRNA-seq data combined with matrix factorization algorithms and machine learning models, we identified multiple APA-associated gene modules linked to ASD and systematically characterized their regulatory patterns across cell types, brain regions, and sexes. To systematically dissect such co-regulatory programs, matrix-factorization-based module discovery methods have advanced rapidly in recent years. Non-negative matrix factorization (NMF) [48] and its sparse variant (sNMF) [49] enable the extraction of functionally coherent co-expression modules from high-dimensional single-cell data, while consensus NMF (cNMF) [50] further enhances module stability through repeated factorization runs followed by clustering. Building upon these approaches, the SMAF method proposed by Cleary et al. [23] is particularly well-suited to our study context: it incorporates an L1 sparsity constraint to highlight core APA-related genes and optimizes the objective function to minimize redundancy among modules, thereby effectively identifying biologically specific APA regulatory programs despite the inherent noise and sparsity of snRNA-seq data. Although SMAF originates from the compressed sensing framework, in this study, we do not employ it for signal reconstruction; rather, we leverage it as an efficient sparse structure learning tool to capture the fine-grained regulatory patterns of APA modules across dimensions of cell type, brain region, and sex. Analyzing the molecular mechanisms of ASD from the perspective of gene modules not only enables the identification of key gene modules involved in specific biological processes but also reveals how APA regulates the functions of these modules, providing new insights into the pathogenesis of ASD.

We observed that APA regulatory landscapes exhibit significant heterogeneity across cell types, with the most pronounced alterations in excitatory neurons (e.g., L2/3 and L5/6), inhibitory neurons (e.g., IN-SST and IN-VIP), and glial cells [8,51,52]. These APA modules are not only significantly enriched for known ASD risk genes (SFARI genes) but also converge on key biological pathways, including neurodevelopment, synaptic transmission, and ion transport. This suggests that APA dysregulation may contribute to ASD by perturbing these critical signaling networks. Notably, despite both being excitatory neurons, L2/3 and L5/6 display opposing APA trends: L2/3 neurons predominantly utilize proximal poly(A) sites, resulting in 3′ UTR shortening, whereas L5/6 neurons favor distal poly(A) site usage, leading to 3′ UTR lengthening. Our identification of 55 high-recurrent APA modules provides novel insights into the molecular architecture underlying this differential susceptibility. We hypothesize that the observed 3′ UTR shortening in L2/3 neurons may lead to an escape from miRNA-mediated repression—specifically through the loss of binding sites for synaptic regulators such as *miR-134* or *miR-125* [53,54,55,56,57]. This mechanism could result in the overexpression of key synaptic proteins like *SYT1* and *SNAP25*, thereby directly contributing to the excitation/inhibition (E/I) imbalance characteristic of ASD. Conversely, the 3′ UTR lengthening in L5/6-CC neurons might reflect an aberrant attempt to increase regulatory complexity or alter the subcellular localization of transcripts critical for long-range projection.

Furthermore, our results point to potential upstream drivers involving RNA-binding proteins (RBPs). Several genes within our ASD-associated modules are known targets of *FMRP* (Fragile X Mental Retardation Protein), a key RBP whose dysfunction is tightly linked to ASD [58]. Since *FMRP* preferentially binds to mRNAs with long 3′ UTRs to regulate their transport and translation at synapses, the widespread APA alterations observed here—particularly the shifts in 3′ UTR length—could disrupt *FMRP* binding landscapes, leading to the mislocalization of synaptic transcripts [59]. Similarly, the dysregulation of other neuro-specific RBPs such as *ELAVL4* (*HuD*) or *HNRNP*s, which govern poly(A) site selection, may underlie the global shifts in 3′ UTR length we observed [60]. Future experimental validation of RBP binding profiles in these specific cell types will be crucial to dissect these mechanisms. Furthermore, we detected a substantial number of differential APA events in Neu-mat, despite the absence of significant changes at the overall gene expression level. This finding underscores APA as a crucial regulatory layer capable of profoundly influencing gene function without altering total transcript abundance, highlighting its underappreciated role in ASD pathogenesis.

Sex differences represent a prominent clinical feature of ASD. Our analysis reveals marked sex-specific APA regulatory patterns. For instance, in the L2/3_3_10 module, male-associated genes are enriched in synaptic functions, while female-associated genes are more involved in energy metabolism regulation [61,62]. These sex-specific patterns offer a compelling mechanistic explanation for the male bias in ASD prevalence. The enrichment of mitochondrial metabolism pathways in females suggests a compensatory APA reprogramming that enhances bioenergetic capacity to buffer against synaptic deficits. In contrast, males exhibit predominant synaptic APA dysregulation without this metabolic buffer, rendering them more vulnerable to synaptic dysfunction. This “metabolic compensation” hypothesis via APA modulation provides a new avenue for understanding sexual dimorphism in neurodevelopmental disorders [63,64]. These findings emphasize the importance of considering sex-specific post-transcriptional regulation in future mechanistic and therapeutic studies.

At the regional level, we identified significant differences in APA regulation between ACC and PFC. For example, in the AST-PP_2_4 module, ACC-enriched genes are involved in glutamate homeostasis, whereas PFC-enriched genes primarily regulate synaptic excitability. This region-specific APA landscape may underlie the phenotypic heterogeneity of ASD: APA alterations in the ACC may disrupt emotional and social motivation circuits, contributing to social deficits, while APA dysregulation in the PFC may exacerbate cognitive inflexibility through heightened synaptic excitation. These observations reinforce the role of APA as a spatially resolved regulatory mechanism in distinct neuropathological processes.

In addition, we evaluated the utility of APA modules in disease prediction modeling. XGBoost-based classification models demonstrated strong performance using APA data across multiple cell types. Moreover, integrating APA with gene expression data further improved prediction accuracy, indicating that APA provides independent and complementary biological signals. This not only validates the diagnostic potential of APA in complex disorders but also establishes a methodological foundation for developing multimodal, precision prediction models for ASD.

The findings of this study reveal a novel mechanism underlying the heterogeneity of ASD across different human brain cell types. By systematically dissecting cell type-, brain region-, and sex-specific APA landscapes, we have identified multiple ASD-associated gene modules enriched for SFARI risk genes and involved in critical neurodevelopmental pathways. Compared to conventional single-cell transcriptomic analyses, our APA-based, cell-type-specific gene network approach offers unique advantages in elucidating disease mechanisms, even in the absence of single-cell data from affected individuals. This study provides new insights into the molecular pathology of ASD and establishes a broadly applicable framework for investigating other complex diseases.

Despite providing novel insights into the role of APA in ASD, this study has several limitations. First, our inference of APA regulatory patterns is based on postmortem brain snRNA-seq data, which cannot capture the dynamic, developmental trajectory of APA dysregulation across disease progression. Second, to prioritize high-confidence genetic mechanisms, we filtered modules based on enrichment for SFARI risk genes [6,7]; this approach may have overlooked non-genetic APA events driven by environmental factors or neuroimmune responses—such as inflammation-related regulation in microglia. Third, gene expression modules and APA modules were defined using different core gene selection criteria: the former employed Z-score thresholds with an upper size limit (<800 genes), whereas the latter relied on *t*-test-based significance without size constraints. Consequently, the observation that “APA modules contain more genes” may partly reflect methodological differences rather than a true biological phenomenon. Finally, the binary thresholding strategy used to define core genes does not fully leverage the probabilistic, continuous nature of gene-module membership inherent in factorization models; future work will benefit from integrating Grade of Membership (GoM)-based approaches (e.g., fastTopics [65]) to better capture the multifaceted roles of genes across regulatory programs. Nonetheless, our analytical framework establishes a valuable foundation for dissecting post-transcriptional regulatory heterogeneity in complex neurodevelopmental disorders.

## 4. Materials and Methods

### 4.1. Data and Preprocessing

We used human brain snRNA-seq data from the study by Velmeshev et al. (2019) [8]. The dataset includes 41 postmortem brain tissue samples from 15 individuals with ASD and 16 healthy human controls, covering the PFC and ACC. This dataset comprises a total of 104,559 nuclei, annotated into 17 major cell types: fibrous astrocytes (AST-FB), protoplasmic astrocytes (AST-PP), endothelial, parvalbumin interneurons (IN-PV), somatostatin interneurons (IN-SST), SV2C interneurons (IN-SV2C), VIP interneurons (IN-VIP), layer 2/3 excitatory neurons (L2/3), layer four excitatory neurons (L4), layer 5/6 corticofugal projection neurons (L5/6), layer 5/6 cortico-cortical projection neurons (L5/6-CC), microglia, maturing neurons (Neu-mat), *NRGN*-expressing neurons I (Neu-*NRGN*-I), *NRGN*-expressing neurons II (Neu-*NRGN*-II), oligodendrocytes, and oligodendrocyte precursor cells (OPCs). Details of the cell types are provided in Table S1.

Raw data were obtained from the GEO (Gene Expression Omnibus) database and preprocessed using the scran v1.38.1 package in R, including quality control of nuclei and genes, removal of a small number of nuclei from distinct cell cycle stages, and normalization of gene expression data [66]. To eliminate the impact of sequencing technical factors and potential biological confounders (including sex, age, postmortem interval, and RNA integrity) on gene expression heterogeneity, regression correction was performed using the ComBat method, followed by filtering of nuclear genes and mitochondrial genes from Human MitoCarta2.0 [67]. Ultimately, high-quality expression data from 76,244 nuclei were retained for subsequent analyses.

### 4.2. Identification and Quantification of Poly(A) Sites

We employed scAPAtrap [68] to identify and quantify genome-wide poly(A) sites from snRNA-seq data, followed by annotation and quality control using movAPA [69]. The detailed workflow proceeded in three key steps:

Step 1: Initial Site Selection. We retained all initial poly(A) sites detected in at least 10 cells and supported by ≥10 sequencing reads as the candidate site set.

Step 2: Two-Stage Filtering for Internal Priming (IP). To effectively mitigate technical false positives, we implemented a rigorous two-stage strategy. First, for sequence-based preliminary identification, we used the removePACdsIP function of movAPA to scan the genomic sequence within a ±50 bp window around each candidate site. Sites were flagged as potential IP artifacts if they exhibited any of the following features: (a) a stretch of ≥6 consecutive adenines (A-tract); (b) dense A/T-rich regions (e.g., AAAAAT, TTTTTA); or (c) absence of canonical poly(A) signals, such as AAUAAA or its common variants. Second, for validation and rescue, we cross-referenced all “IP-flagged” sites with two authoritative databases: GENCODE v44 and polyA_DB3. Any site overlapping (within ±10 bp), a known poly(A) site in these databases was reclassified as a “validated true site.” Ultimately, we merged non-IP sites and rescued IP sites for downstream analysis, a strategy that rigorously controls false positives while maximizing the retention of biological signals.

Step 3: Annotation and RUD Calculation. Retained sites were annotated using the TxDb.Hsapiens.UCSC.hg38.knownGene v3.22.0 package. We strictly retained only those sites located within the 3′ UTR of protein-coding genes to ensure measured events reflect genuine regulatory variation. For each gene, we computed the Relative Usage of Distal polyA site (RUD) metric. RUD is defined as the proportion of reads mapped to the most distal site relative to the total reads of all 3′ UTR sites, calculated as

(1) $${RUD}_{gi}=\frac{P_{g,i,T_{g}}}{\sum_{t=1}^{T_{g}}\;P_{g,i,t}}$$

where $P_{g,i,t}$ denotes the read count of the t-th site for gene g in cell i, and Tg is the index of the most distal site. A higher RUD value indicates a preference for distal site usage (longer 3′ UTR). By systematically computing RUD for each gene in each cell, we constructed the final RUD matrix (cells × genes) for downstream analysis.

### 4.3. Sparse Matrix Factorization

For each cell type, its expression matrix $X\in R^{G\times N}$ (where G represents the number of genes and N represents the total number of cells) was randomly and independently divided into a training set (70%) and a test set (30%). This partitioning was repeated 10 times to generate 10 independent training sets ($X_{train}^{\left(i\right)}$, where i = 1,…,10).

In this study, the SMAF algorithm [23] was applied to the 10 RUD matrices of each cell type’s training set to identify APA gene modules. The factorization is defined as

(2) $$X_{trn}\approx UW$$

Here, $U\in R^{G\times d}$ is a non-negative sparse module dictionary, where each column represents a gene module and each row indicates the weight of a specific gene within that module. $W\in R^{d\times n}$ is the module activity matrix (where n is the number of cells in the training set); each column represents a cell, and each row represents the activity score of a specific module across cells.

SMAF imposes dual sparsity constraints on both U and W. This ensures that the identified modules exhibit distinct, highly gene-specific APA patterns with minimal overlap, aligning with the intrinsic sparsity of single-nucleus APA data.

To comprehensively explore the solution space, we set the initial number of modules (rank) to d=500 for all cell types. This “over-complete dictionary” strategy allows the algorithm to capture potential fine-grained patterns without artificially limiting the search space. Given the strict sparsity constraints (k=15 for W, limiting active modules per cell; $\lambda_{da2}\;=0.1$ for U, controlling gene weight sparsity), many of the initial 500 modules are redundant or unstable.

To isolate robust biological signals, we implemented a rigorous two-step filtering process:

(1) Stability Filtering: We calculated the recurrent rate (Jaccard similarity coefficient) for each of the 500 modules across the 10 independent runs. Only modules with a recurrent rate ≥ 80% (or ≥75% for cell types yielding fewer than 5 stable modules) were retained as high-confidence candidates.
(2) Biological Relevance Screening: On these stable modules, we performed statistical testing (e.g., *t*-tests on module activities between ASD and control groups) to identify those significantly associated with ASD.

This strategy ensures that the final reported modules (e.g., the 55 high-confidence ASD-associated modules) are both mathematically robust and biologically meaningful.

### 4.4. Identification of APA-Associated Gene Modules

To identify gene modules associated with ASD through APA, we applied a two-step statistical screening procedure.

First, for each module m, we extracted its poly(A) site selection propensity across all cells as a vector $W_{m,*}\in R^{1\times s}$. Cells were partitioned into ASD ($W_{a}=\left[W_{m,1},\ldots ,W_{m,s_{0}}\right]$) and control ($W_{c}=\left[W_{m,s_{0}+1},\;\ldots ,W_{m,s}\right]$) groups. We performed a Wilcoxon rank-sum test to assess whether the module’s activity differed significantly between the two groups. *p*-values were adjusted using the Benjamini–Hochberg procedure to control the false discovery rate (FDR); modules with an adjusted *p*-value < 0.01 were retained.

Second, we evaluated the strength and significance of the association between module activity and disease status. Sample labels were encoded as a binary vector $Y=[y_{1},\;\ldots ,\;y_{s}]$, where $y_{k}=+1$ for ASD and $y_{k}=-1$ for control. The Spearman rank correlation coefficient $\rho_{m}$ between $W_{m,*}$ and Y was computed. To assess significance without relying on asymptotic assumptions, we used a permutation test (1000 permutations) to derive empirical *p*-values, which were then FDR-corrected. Modules were considered meaningfully associated with ASD if they met both: $\left|\rho_{m}\right|>0.1$ (a minimal effect size threshold) and an adjusted *p*-value < 0.01.

Only modules satisfying both criteria—significant group difference (Wilcoxon) and robust label correlation (Spearman + permutation)—were classified as APA-associated. While these two tests are statistically related under binary grouping, their combination serves to jointly enforce statistical significance and biological relevance (via effect size filtering), thereby reducing false positives driven solely by large sample sizes or negligible effects.

### 4.5. Core Gene Selection Within Modules and Module Robustness Assessment

To identify core genes within each APA-associated gene module derived from the SMAF of the RUD matrix, we employed a Z-score-based adaptive thresholding strategy on the module dictionary matrix (U). In this matrix, each entry represents the loading weight of a specific gene in a given module.

First, for each module (column in U), we calculated the Z-score for the loading weights of all genes to normalize their contributions relative to the module’s background distribution. Genes with higher Z-scores indicate stronger specificity and contribution to that particular module.

Second, to ensure the biological interpretability of the modules and avoid overly broad gene sets, we implemented an iterative threshold adjustment procedure. We initialized the Z-score threshold at 1.0 and incrementally increased it by steps of 0.05. This process continued until the number of selected genes for each module was reduced to fewer than 1000. The genes exceeding this final adaptive threshold were designated as module-specific core genes. This data-driven approach ensures that each module is represented by a concise set of highly specific genes, facilitating downstream functional enrichment analysis.

To mitigate the uncertainty introduced by the random initialization inherent in matrix factorization, the “recurrent rate across data blocks” was introduced as a key metric for evaluating module robustness. Given the heterogeneity of cell types, recurrence rates were computed only within the same cell type across different training subsets. For each cell type, ten data blocks were generated. For any two distinct blocks n and o (n≠o) if the number of genes in the i-th module $M_{n,i}$ of block n is $m_{n,i}$, then the maximum recurrence rate of this module in block o is

(3) $${Mrr}_{n,i,o}=max\left[\frac{M_{n,i}\cap M_{o,1}}{m_{n,i}},\ldots ,\frac{M_{n,i}\cap M_{o,K_{o}}}{m_{n,i}}\right]$$

where $j\in \left\{1,\;2,\;\ldots ,\;Ko\right\}$ indexes all modules in block o. This metric quantifies the proportion of shared genes between modules and reflects the stability of modules across different data partitions.

Finally, a recurrence rate threshold was applied to identify highly stable APA gene modules. By default, a threshold of 80% was used to define module stability; however, for cell types with fewer modules (e.g., Neu-mat), the threshold was slightly relaxed to 75%. Modules meeting this recurrence criterion were considered robust and were retained as high-confidence ASD-associated APA gene modules for downstream functional analysis and biological interpretation.

### 4.6. Functional Enrichment Analysis of Modules

To identify APA gene modules significantly associated with ASD and exhibiting cell type specificity, this study conducted a two-stage gene enrichment analysis. First, hypergeometric enrichment tests were performed to assess whether the overlap between each APA gene module and two gene sets was statistically significant: the marker genes of its corresponding cell type and a set of known ASD candidate genes. If a module showed significant enrichment with either the cell type marker genes or the ASD-associated genes, it was considered to exhibit cell type specificity or a significant association with ASD. Cell type marker genes were identified using the findMarkers function of scran applied to the original gene expression data, and the top 100 differentially expressed genes for each cell type were selected as its characteristic genes. ASD-associated genes were obtained from the SFARI database [6,7].

Second, functional annotation analysis was performed on the screened ASD-related APA gene modules. The R package clusterProfiler v4.18.4 was used to conduct GO functional enrichment and KEGG pathway analysis on module genes, and functional networks and pathway networks were constructed to reveal the potential biological processes and signaling pathways they participate in. To further explore the role of these modules in molecular mechanisms, a PPI network was built based on genes within the modules. The list of module genes was input into the STRING database to obtain potential interaction relationships, and Cytoscape v3.10.4 was used for visual analysis of the PPI network [70]. The connectivity (degree) of each gene in the network was calculated using the NetworkAnalyzer v4.5.0 plugin to identify key genes playing a core regulatory role in the network, namely hub genes.

### 4.7. Identification of Sex-Specific and Brain Region-Specific Modules

To identify APA regulatory patterns with sex- or brain region-specific characteristics, this study further evaluated the previously identified ASD-associated, cell-type-specific APA gene modules for differences in poly(A) site usage preference between sexes (male/female) and brain regions (PFC/ACC). This assessment employed the sciRED tool [35] in conjunction with the two statistical methods described earlier (Wilcoxon rank-sum test and Spearman correlation analysis).

Specifically, sciRED was utilized to assess potential associations between APA modules and covariates (such as sex and brain region) [35]. By inputting the module’s W matrix and sample labels into the model, FCA scores were computed to identify APA modules exhibiting sex- or brain region-specificity. Furthermore, for the identified APA modules, differential APA events between disease and healthy states were calculated using the limma v3.66.0 [71] package based on their corresponding RUD matrices, thereby revealing their dynamic changes under pathological conditions.

### 4.8. Construction of ASD Prediction Models Based on APA Modules

To avoid data leakage and ensure the biological validity of our model evaluation, all machine learning analyses employed a donor-level train-test split strategy. Specifically, cells were first grouped by their donor_id from the metadata. Each donor’s entire set of cells was treated as an indivisible unit and randomly assigned in its entirety to either the training set or the test set (at a 7:3 ratio). This ensures that no single donor contributed cells to both sets, thereby preventing the model from learning donor-specific artifacts (e.g., batch effects or genetic background) and truly evaluating its ability to generalize to new individuals with ASD.

To evaluate the application value of APA modules in the classification and prediction of ASD, this study constructed two machine learning models: an XGBoost model based on a single data source and a PSVM-2V [72,73].

The XGBoost algorithm used APA gene modules in the RUD matrix and gene modules in the gene expression matrix as features, respectively, to build cell-type-specific ASD classification models. By comparing the model performance across different cell types, the potential biological significance of these modules in disease mechanisms was revealed. The model was implemented using the R package XGBoost v2.1.0 with the following parameter settings: maximum tree depth of 6, learning rate of 0.01, training sample ratio of 0.5, and number of iterations set to 10.

The PSVM-2V fused information from APA modules and gene expression modules to further improve classification performance. This method not only integrates the shared features of the two data sources but also fully explores the unique information contained in each modality. During training, PSVM-2V maximizes classification accuracy by weighted fusion of information from different views and introduces a weight coefficient $\beta_{v}$ (ranging from 0 to 1) to quantify the relative contribution of each modality to the classification result.

## Data Availability

The data presented in this study are openly available in the NCBI Sequence Read Archive (SRA) under BioProject accession PRJNA434002 (Velmeshev et al., 2019) [8], and the analysis pipeline is openly available at: https://github.com/BMILAB/ASD-module (accessed on 2 March 2026).

## Supplementary Materials

The following supporting information can be downloaded at: https://www.mdpi.com/article/10.3390/ijms27062849/s1.

## Abbreviations

ASD	autism spectrum disorder
APA	alternative polyadenylation
snRNA-seq	single-nucleus RNA sequencing
RBP	RNA-binding protein
miRNA	microRNA
3′ UTR	3′ untranslated region
SFARI	Simons Foundation Autism Research Initiative
m^6^A	N^6^-methyladenosine
SMAF	sparse module activity factorization
PAS	polyadenylation signals
UMAP	uniform manifold approximation and projection
RUD	relative usage of the distal poly(A) site
KEGG	Kyoto Encyclopedia of Genes and Genomes
PPI	protein–protein interaction
FCA	factor–covariate association
GO	Gene Ontology
PFC	prefrontal cortex
ACC	anterior cingulate cortex
XGBoost	eXtreme Gradient Boosting
PSVM-2V	Multi-view Privileged Support Vector Machine
NMF	non-negative matrix factorization
sNMF	sparse non-negative matrix factorization
cNMF	consensus non-negative matrix factorization
GoM	grade of membership
GEO	Gene Expression Omnibus
FDR	false discovery rate
AST-FB	fibrous astrocytes
AST-PP	protoplasmic astrocytes
IN-PV	parvalbumin interneurons
IN-SST	somatostatin interneurons
IN_SV2C	SV2C interneurons
IN_VIP	VIP interneurons
L2/3	layer 2/3 excitatory neurons
L4	layer four excitatory neurons
L5/6	layer 5/6 corticofugal projection neurons
L5/6-CC	layer 5/6 cortico-cortical projection neurons
Neu-mat	maturing neurons
Neu-*NRGN*-I	*NRGN*-expressing neurons I
Neu-*NRGN*-II	*NRGN*-expressing neurons II
OPC	oligodendrocyte precursor cell
