# Supplementary material for: Single-Cell Gene Module Inference Reveals Alternative Polyadenylation Dynamics Associated with Autism
Source: Int J Mol Sci. 2026 Mar 21;27(6):2849. doi: 10.3390/ijms27062849 (PMC13027334; doi:10.3390/ijms27062849)
Supplement: Supplementary file 1 [file ijms-27-02849-s001.zip › ijms-4126192-Supplementary Figures.pdf]

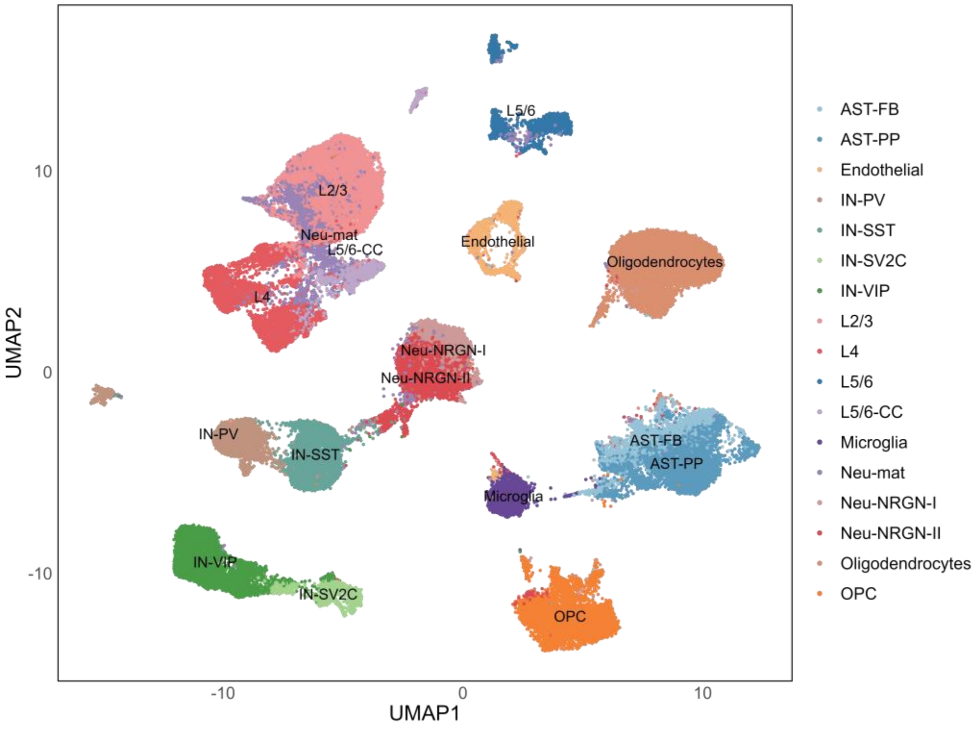

Figure S1. UMAP visualization based on single-cell gene expression profiles.

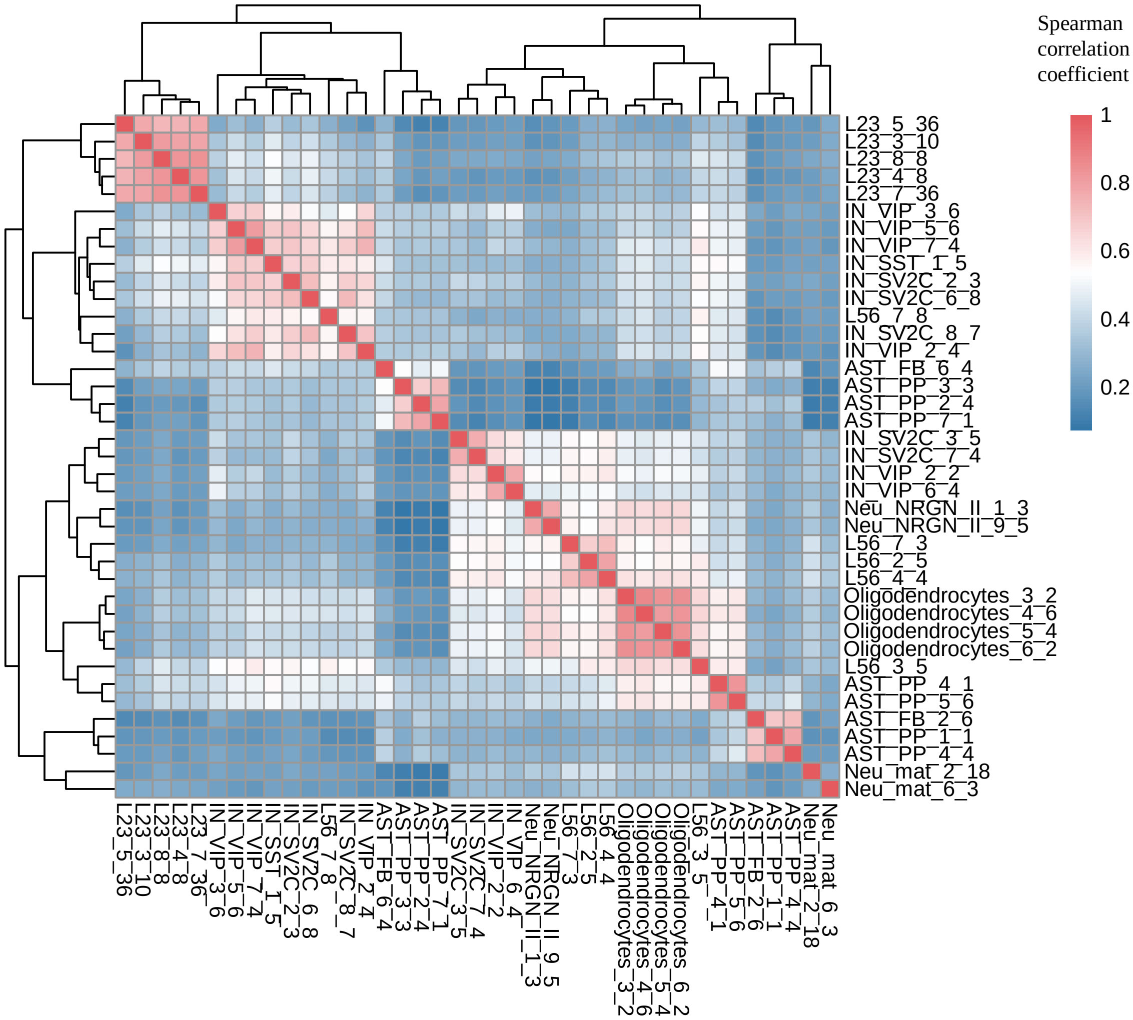

Figure S2. Spearman correlation analysis among APA gene modules.

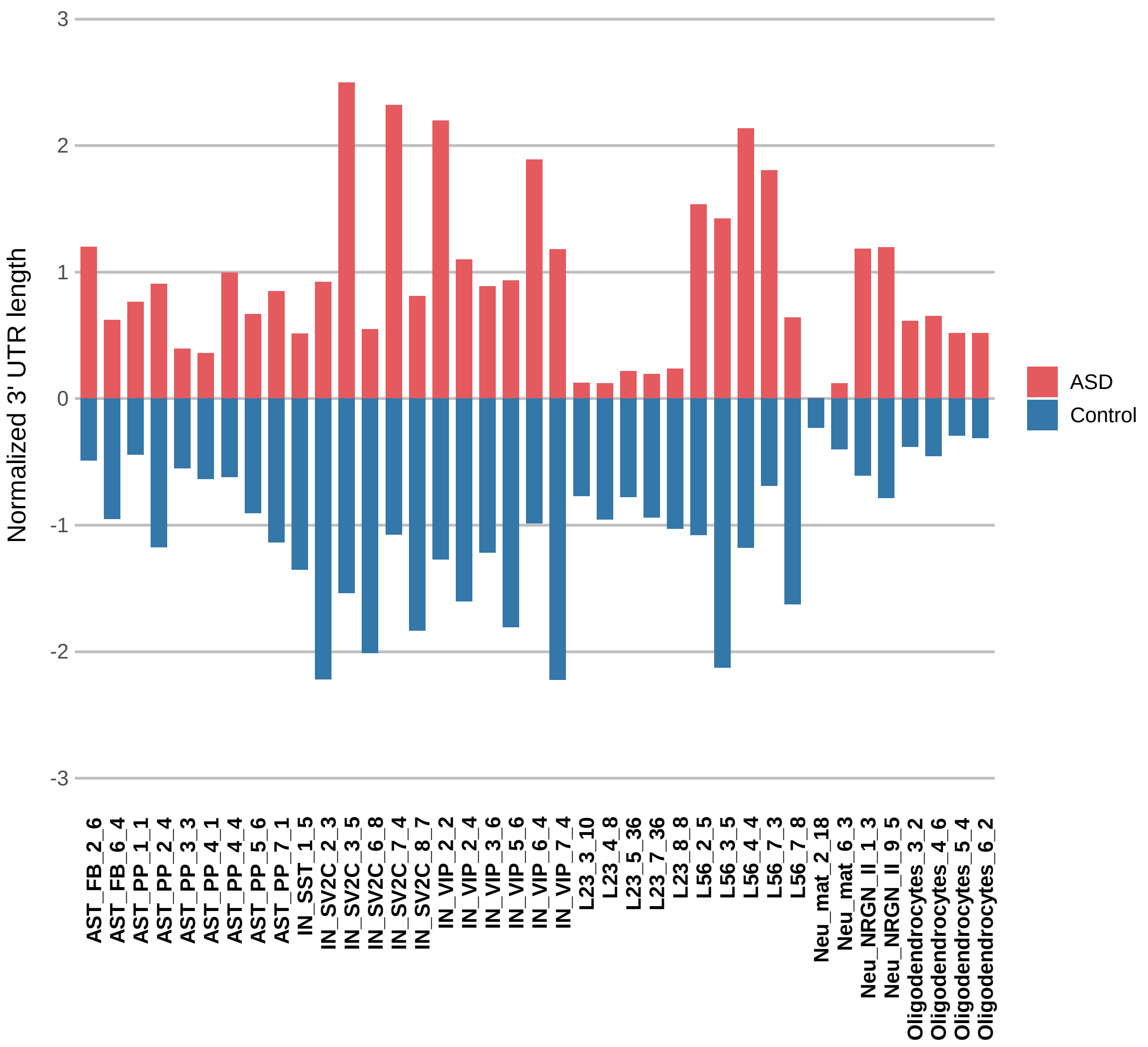

Figure S3. Comparison of 3' UTR length in cell type-specific APA modules between ASD and control groups.

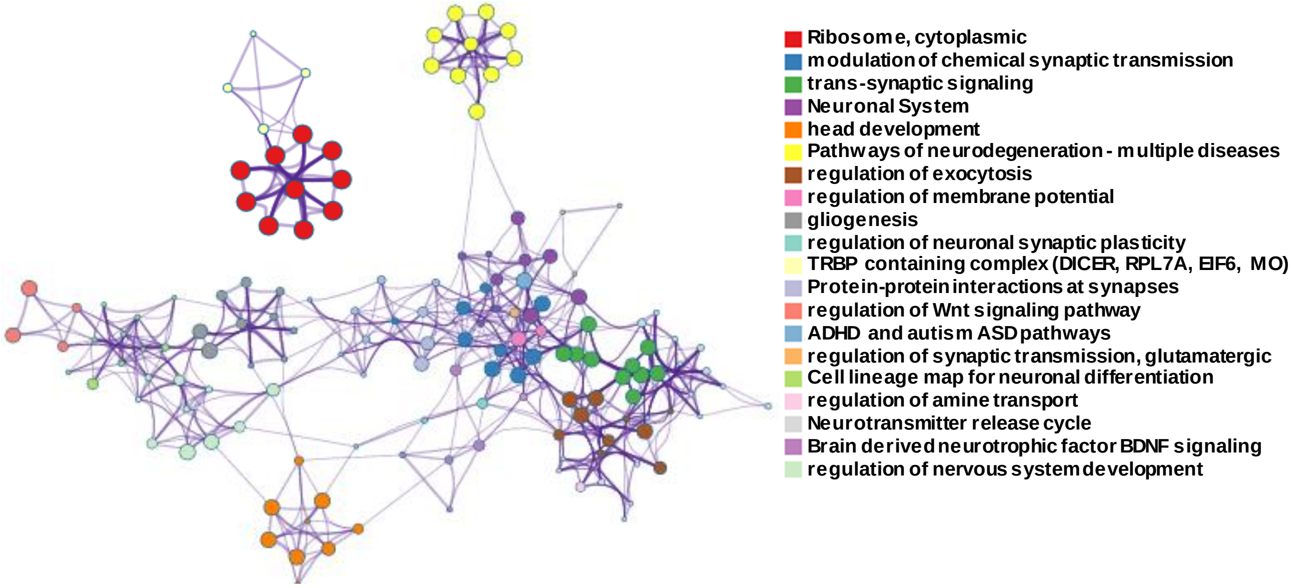

Figure S4. Functional enrichment analysis of APA modules. Each node represents a functional category; the size of the node reflects the degree of enrichment of that functional category within the APA modules; connecting edges indicate the interrelationships among functional categories, with thicker lines representing stronger associations.

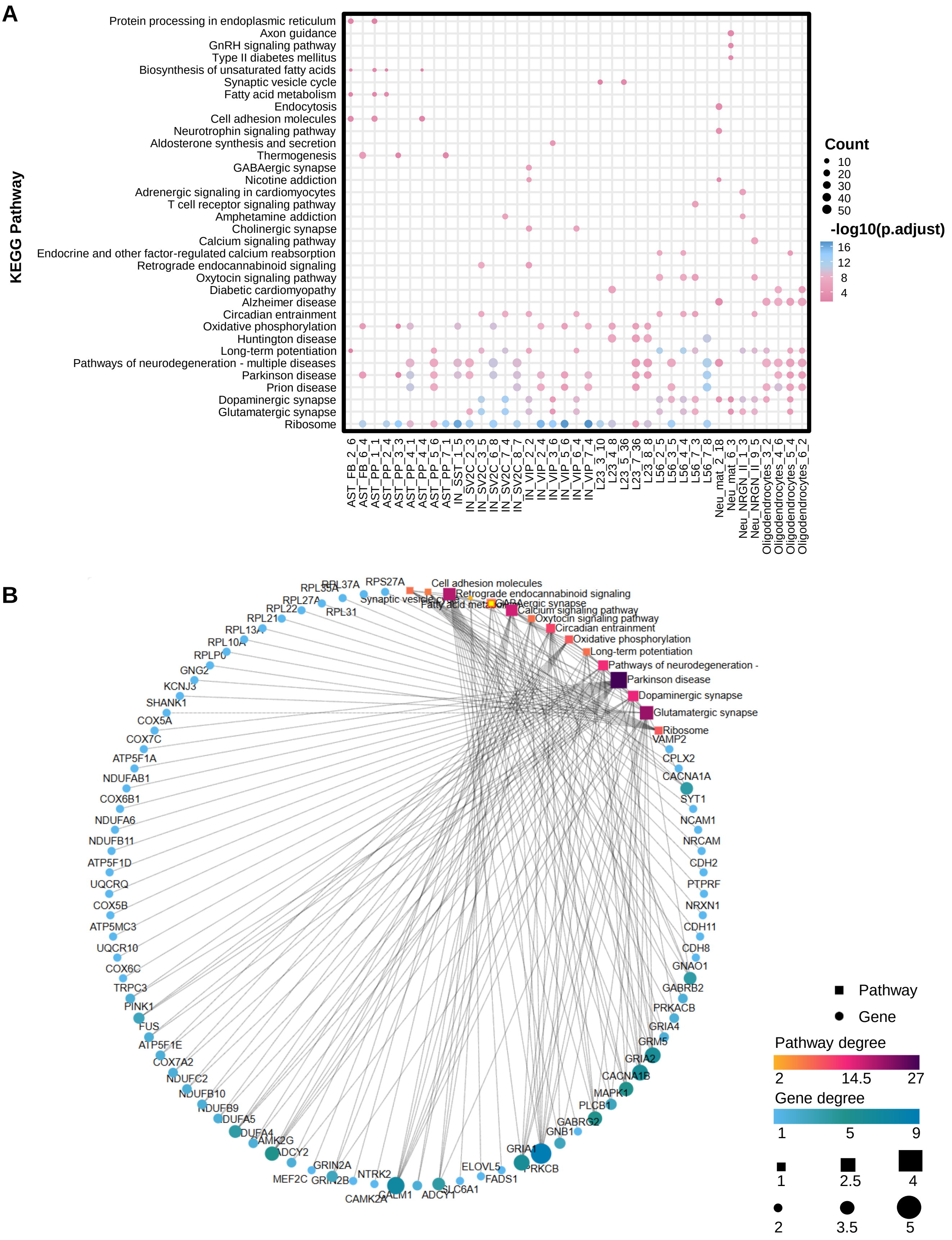

Figure S5. Pathway enrichment analysis of APA modules. (A) APA regulation is involved in the pathogenesis of ASD by affecting multiple neurosystem signaling pathways and metabolic processes. (B) Cooperative roles of these pathways in the pathological process of ASD.

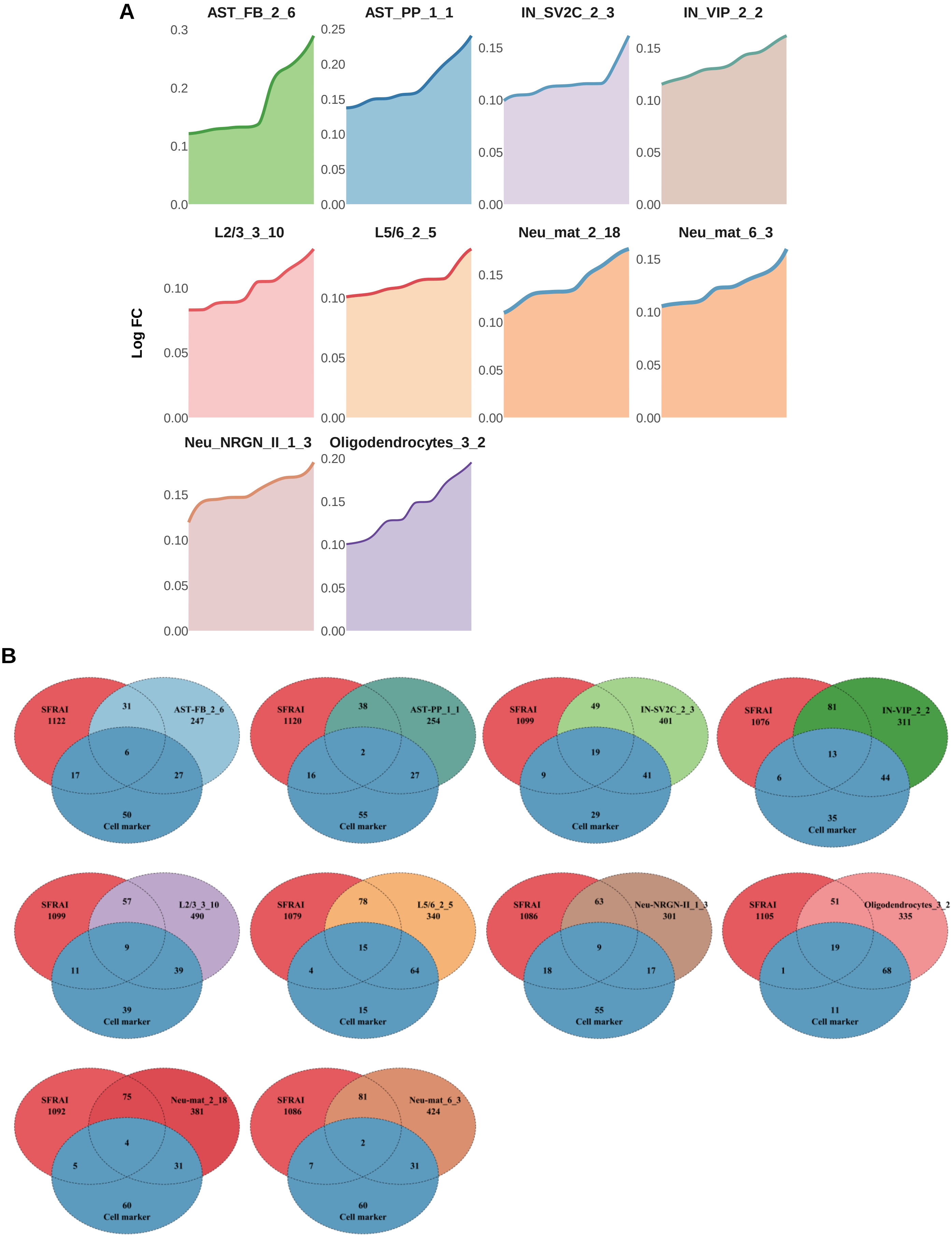

Figure S6. Molecular characterization analysis of differentially expressed APA genes in ASD versus control groups. (A) Visualization of the top 10 differentially expressed APA genes in each module, ranked by $\log_2$ fold change (Log_2 FC) along the x-axis in ascending order. (B) Venn diagrams showing the gene overlap among APA module genes, SFARI genes, and cell type-specific marker genes.

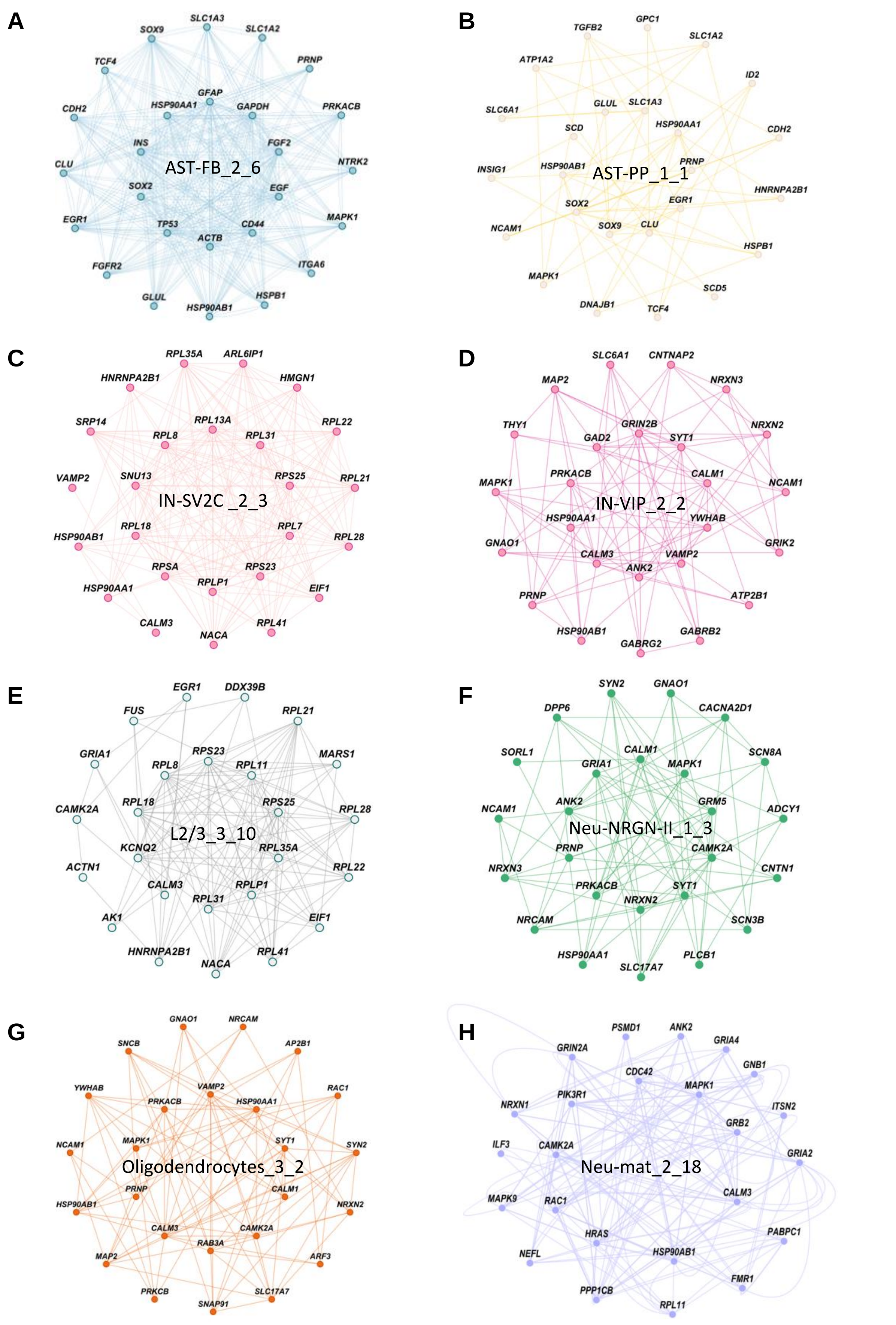

Figure S7. Protein-protein interaction (PPI) networks constructed based on hub genes of APA modules. Each PPI network corresponds to a specific APA module.

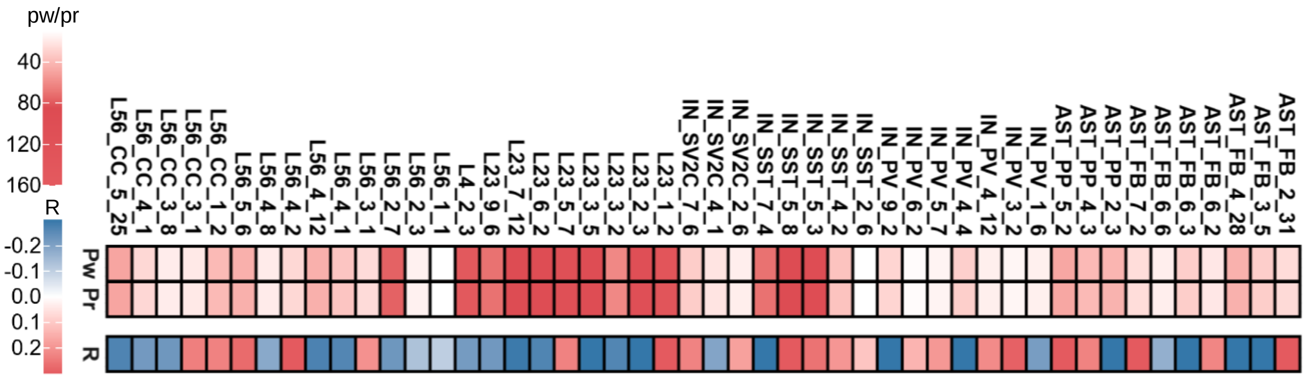

Figure S8. Heatmap of ASD-related tests for 48 gene expression modules with a recurrent rate greater than 80%. The “Pw” and “Pr” rows represent the -log10 adjusted *P*-values from the Wilcoxon test and Spearman test, respectively; the “R” row indicates the Spearman correlation coefficient. Each gene expression module is identified by a code comprising its cell type, dataset chunk number, and the sequence number of the module within that chunk. For example, APA gene module L2/3_1_1 denotes the first module in the first dataset chunk for L2/3 cell types

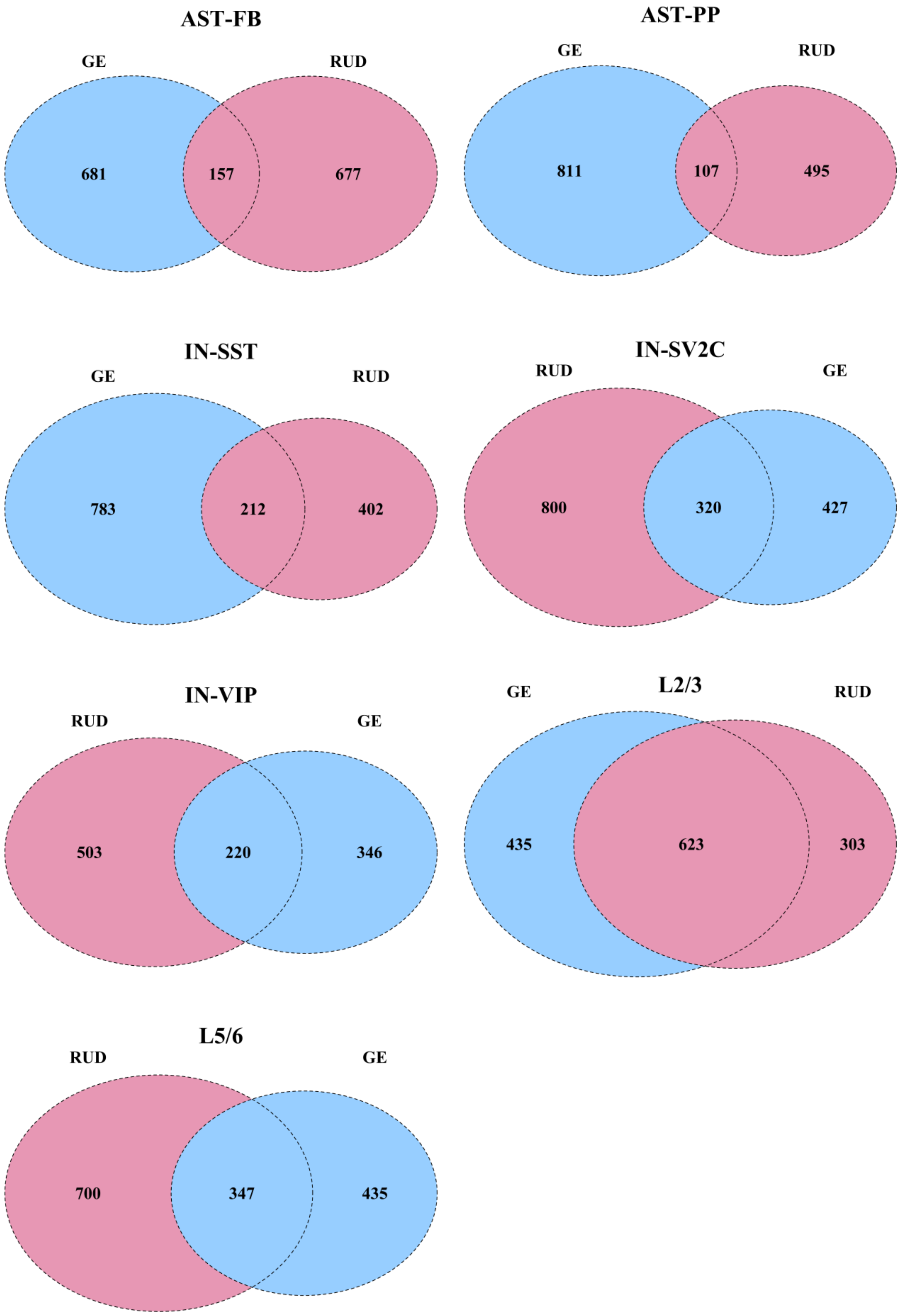

Figure S9. Venn diagrams showing overlapping of genes between APA modules (RUD) and gene expression modules (GE) in shared cell types.
